# Evaluation of human cytomegalovirus antigen expression in invasive breast carcinoma in a population of Iranian patients

**DOI:** 10.1186/s13027-017-0148-3

**Published:** 2017-06-26

**Authors:** Fereshteh Mohammadizadeh, Fatemeh Mahmudi

**Affiliations:** Department of Pathology, School of Medicine, Isfahan University of Medical Sciences, Isfahan, Iran

**Keywords:** Breast cancer, Human cytomegalovirus, Immediate early antigen, Iran

## Abstract

**Background:**

The role of human cytomegalovirus (HCMV) in the development of breast carcinoma is questionable. The aim of this study was to evaluate the expression of the immediate early antigen (IE) of HCMV in breast carcinoma and its association with some clinicopathologic factors in a population of Iranian patients.

**Methods:**

Formalin-fixed and paraffin-embedded tissue blocks from the pathology laboratories of the Azahra and Shahid Beheshti hospitals, Isfahan, Iran, from 2013 to 2016, were used in the study. We used immunohistochemistry and real-time PCR to detect the IE-antigen of HCMV in breast carcinoma, normal tissue adjacent to carcinoma, and normal tissue from mammoplasty specimens.

**Results:**

A total of 96 samples were evaluated: 70 invasive breast carcinoma of different histologic subtypes and 26 mammoplasty normal breast tissues. All the samples were negative for IE-antigen expression. No relationship was seen between breast cancer and HCMV in this study.

**Conclusions:**

The results of this study failed to show any relationship between HCMV and the development of breast carcinoma.

## Background

Breast carcinoma is the second-most-common cause of cancer-related death among women worldwide. However, breast cancer-related death has decreased in recent years due to early detection and treatment [1]. Breast cancer is the most common type of cancer among Iranian women, according to official statistics from the cancer registry [2].

Some of the documented risk factors of breast carcinoma include early age of menarche, late age of menopause, a positive family history of breast cancer, hormone replacement therapy, age, sex, and nulliparity [3–8]. At present, the etiologic role of viral infections in some human cancers—for example, the role of hepatitis B and C viruses in hepatocellular carcinoma, and the role of the Epstein–Barr virus (EBV) in nasopharyngeal carcinoma—has been confirmed [9, 10]. Although several studies led by molecular and protein technologies have detected some viruses, such as the human papillomavirus (HPV) [11, 12] and the EBV [13–15] in breast cancer specimens, findings regarding the human cytomegalovirus’s (HCMV) role in breast cancer is controversial. It has been posed that breast carcinoma may be associated with late exposure to a common virus [16]. Breast cancer incidence has been shown to be low in countries where HCMV seroconversion occurs in childhood, and most adults are HCMV seropositive [14]. On the other hand, breast cancer is most prevalent in those countries where there is late exposure to common viruses [14].

HCMV is a member of the herpes virus family, which can remain latent lifelong after the primary infection and shows recurring activation [7]. It has some gene products with dysregulatory effects on the proliferative cell cycle, resulting in a block of the apoptotic pathways, tumor suppressor proteins dysfunction, and DNA mutation [17, 18]. Consequently, the cells infected by CMV are prone to DNA instability and neoplastic transformation [19]. HCMV has been found in the milk of infected women [3]. So, the virus may have the potential to spread to the adjacent breast epithelial cells [3]. Moreover, HCMV has been found to infect the macrophages and induce an atypical phenotype of M1/M2 macrophages. This phenotype of macrophage releases cytokines, which are involved in cancer initiation or promotion [3].

Since the glandular epithelium of breast tissue can act as a reservoir of latent CMV infection [7], the aim of this study is the evaluation of the probable role of HCMV in the development of breast cancer in a population of Iranian patients, and the possible association between the presence of the virus in carcinoma tissue and some prognostic clinicopathologic parameters of the cancer.

## Methods

Formalin-fixed and paraffin-embedded breast tissue blocks archived in the pathology laboratory of the Azahra and Shahid Beheshti hospitals, Isfahan, Iran, from 2013 to 2016, were used. All of them were previously evaluated for at least ER, PR, and Her2/neu IHC markers. The ethics and scientific research committee of the Isfahan University of Medical Sciences approved the protocol. We had two groups of specimens. The first group included invasive breast carcinoma tissue (as tumor) and adjacent non-neoplastic normal breast tissue (as the tumor control); the second group included normal breast tissue from reduction mammoplasty specimens (as the normal control). The total sample number was 96, including 70 neoplastic (tumor specimens) and adjacent non-neoplastic breast tissue specimens (tumor control), and 26 normal breast tissue specimens (normal control).

Four micron sections were prepared from the tissue blocks and stained by the immunohistochemistry method (IHC) for immediate early antigen (IE) of HCMV. The used antibody was a monoclonal mouse antibody, subclass IgG1, kappa, clone CCH2 (Dako Company, Denmark).

The IHC was carried out as follows: Sections were incubated in an oven at 37 °C for 48 h and then dewaxated by the use of 100% xylol. Rehydration was done by a series of decreasing concentrations of ethanol (100%, 85%, and 75%) and distilled water. The sections were rinsed in a 10% phosphate-buffered saline (PBS) solution. Next, they were incubated in 10% H2O2 and methanol (1:9) for 30 min to prevent endogenous peroxidase activity. They were rinsed again in a 10% PBS solution and then incubated in a citrate-buffered solution (PH = 6.1) in a microwave (power at 800) for 14 min. After rinsing in 100% PBS, blocking of the endogenous non-specific bindings was done by adding a blocking serum (for 30 min), and drying subsequently. In this step, a primary monoclonal antibody (anti-IE with 1:200 ratio) was added, and the samples were incubated at room temperature for 30 min. The samples were then rinsed in a PBS solution and a secondary broad-spectrum antibody was added (30 min). Horseradish peroxidase-streptavidin and diaminobenzidine (DAB) were added for 30 min and 10 min, respectively. The samples were rinsed in 10% PBS, then dehydrated by increasing concentrations of alcohol (75%, 85%, and 100%), and finally counterstained with hematoxylin, and mounted.

**Fig. 1:**
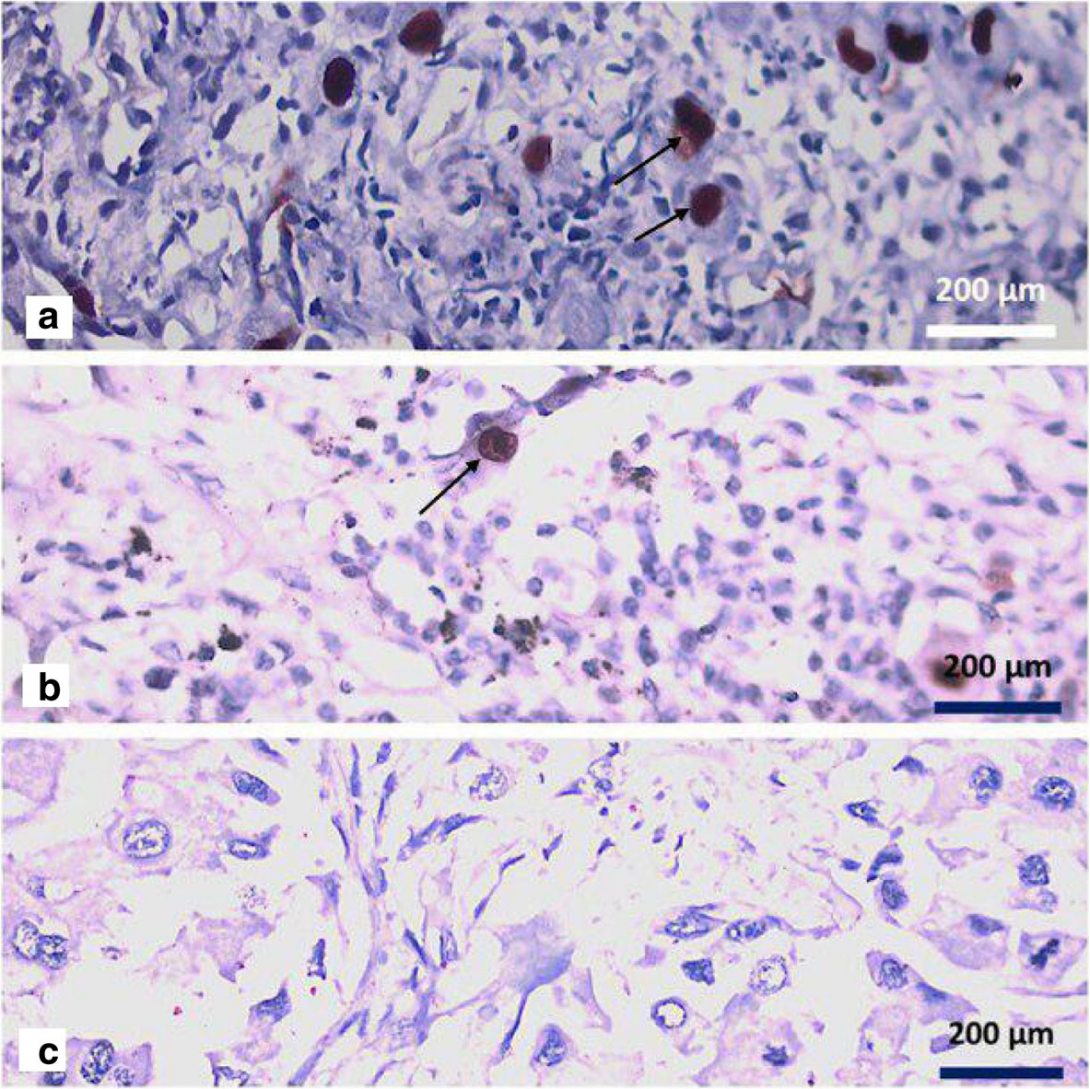
**a** Established HCMV esophagitis in an immunocompromised 49 years old patient, nuclear staining by anti-IE-antigen is seen, marked by arrows. (×40 Objective) (**b**) Established HCMV pneumonia in a 57 years old male, nuclear staining by anti-IE-antigen is seen, marked by arrows. (×40 Objective) (**c**) Negative immunoreactivity for IE-Antigen of HCMV in a tumor sample, in a 45 years old female with grade 3 invasive ductal carcinoma. (×40 Objective)

Two established CMV positive tissue specimens—an esophageal biopsy and a lung biopsy—were also stained concurrently as positive controls Fig. 1a and b .

Finally, two pathologists assessed the IHC-stained slides using a two-headed light microscope.

Nuclear staining with the CMV monoclonal antibody was considered as a positive result, according to the following grading system [7]:

- *Negative: 0*
- *Grade I: < 25% positive cells*
- *Grade II: 25–49% positive cells*
- *Grade III: 50–75% positive cells*
- *Grade IV: > 75% positive cells*

In order to confirm the IHC results, the specimens with positive or equivocal IHC findings were reassessed by real-time PCR. DNA was purified from the tissue blocks using a QIAamp DNA FFPE kit (Qiagen) according to the manufacturer’s instructions. In brief, the process of the DNA purification was as follows: 5–10-μm sections from the paraffin-embedded tissue blocks were prepared. Dewaxation was done by xylene and tissue lysis by proteinase K. Then the sections were incubated at 90 °C to deactivate the proteinase K. DNA binding to the membrane was done, the contaminations were washed, and the DNA was eluted from the membrane.

Finally, real-time PCR was performed with a Corbett Rotor-Gene 6000 analyzer, using a GeneProof CMV PCR kit (the GeneProof CMV PCR kit primer and probes are proprietary and unpublished) to amplify the exon 4 of the IE antigen. Amplification was done with a 10 μl isolated nucleic acid sample and 30 μl Master Mix, as follows: 37 °C/2 min and 95 °C/10 min, and 45 cycles: 95 °C/5 s and 60 °C/40 s, and 72 °C/20 s. All PCR reactions were performed in duplicate.

Other necessary information, including age, tumor size, histologic subtype, histologic grade, and number of involved lymph nodes, were obtained from the patients’ pathology reports. The invasive breast carcinoma was graded according to the Bloom and Richardson grading system.

Analysis was performed using the SPSS20 software (SPSS Inc., Chicago, IL, USA) and independent t-test was used for statistical analysis.

## Results

The characteristics of the samples are presented in Table 1. The independent t-test showed no statistically significant difference between the mean age of the case and control groups (*p* = 0.119) (Table 2).

**Table 1:** Characteristics of the studied samples of invasive breast carcinoma

Sample Characteristics	value (%)
Age (year)	42.47 ± 8.8
Carcinoma type	
Ductal	59 (84.3)
Lobular	6 (8.6)
Mixed Ductal & Lobular	2 (2.9)
Tubular	1 (1.4)
Mixed Ductal & Mucinous	2 (2.9)
Tumor size (cm)	4.4 ± 2.44
Tumor grade in invasive ductal carcinomas	
I	10(15.8)
II	44(69.9)
III	9(14.3)
Nodal Involvement	
None	21(30.0)
1–3	26(37.1)
4–9	17(24.3)
≥10	6(8.6)

**Table 2:** Mean Age of Case and Control Group (year)

	Number	Mean ± SD
Case	70	42.47 ± 8.86
Control	26	39.50 ± 6.14
Total	96	41.47 ± 8.28

Data are presented as mean ± SD or number (%), *SD* Standard deviation

**Fig. 2:**
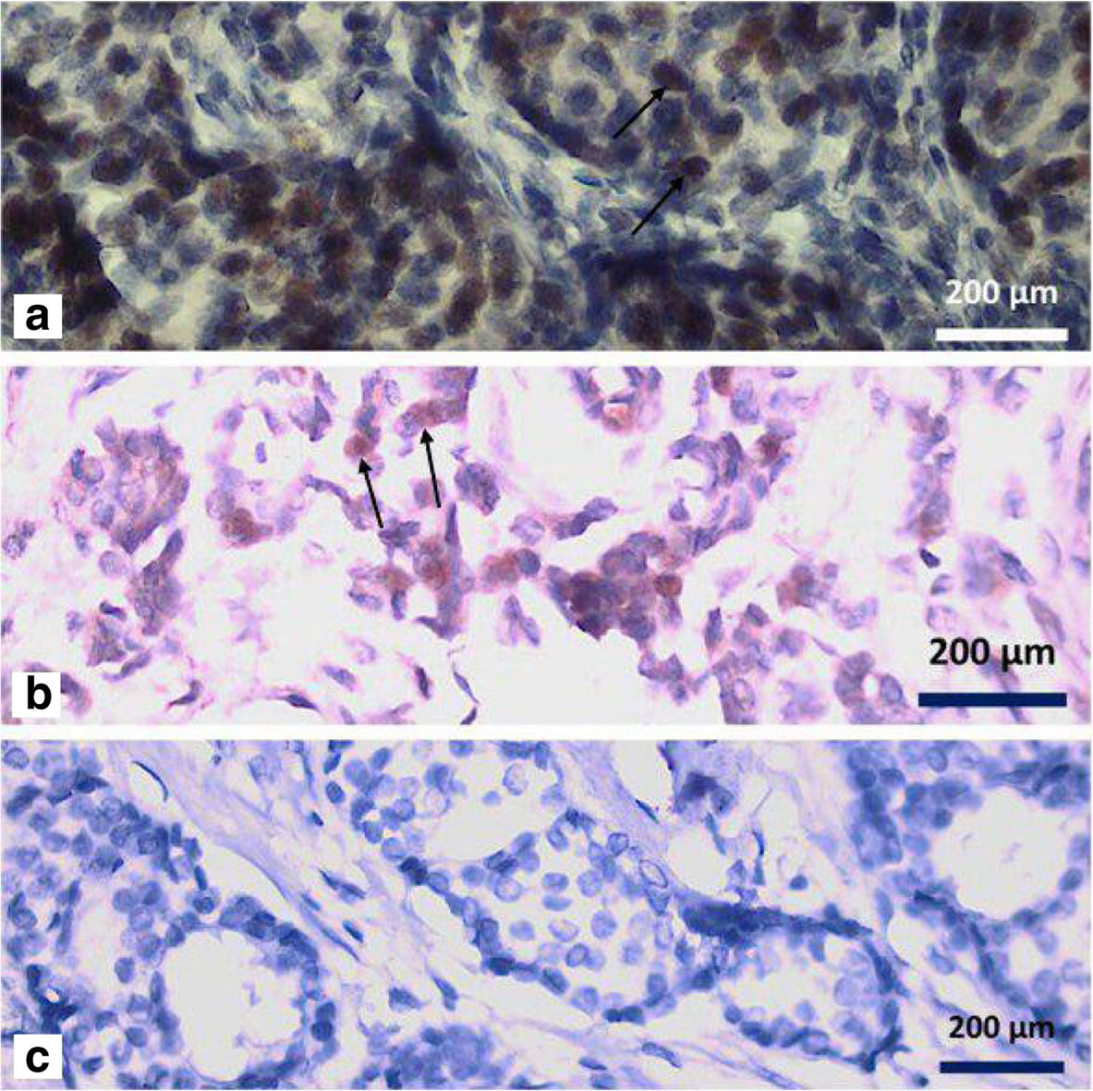
**a** & **b** Two equivocal tumor Samples with weak nuclear staining, marked by arrows: invasive ductal carcinoma, grade 2 in a 34 years old patient (**a**) & invasive ductal carcinoma, grade 2 in a 52 years old patient (**b**). (×40 Objective) (**c**) Negative immunoreactivity for IE-Antigen of HCMV in a tumor sample, in a 44 years old female with grade 1 invasive ductal carcinoma. (×40 Objective)

Fifty-nine of the 70 tumor samples showed invasive ductal carcinoma. Other tumor samples included 6, 1, 2, and 2 invasive lobular, tubular, mixed ductal & lobular, and mixed ductal & mucinous carcinomas, respectively. Furthermore, 15.8% of tumor samples had grade 1 carcinoma, 69.9% had grade 2 carcinoma, and 14.3% had grade 3 carcinoma, according to the Bloom and Richardson grading system. Nodal involvement was seen in 70% of the tumor samples, and 30% of the samples had no nodal involvement. All tumor controls and normal controls were immunohistochemically negative for CMV Fig. 1c and Fig. 2c. Only two tumor specimens showed a double-checked equivocal IHC result Fig. 2a and b. Real-time PCR was done for these two specimens, but CMV DNA was not detected in either of them (Table 3).

**Table 3:** IE-Antigen expression

	IHC			Real time PCR	
	Positive	Negative	Equivocal	Negative	Positive
Case	0	68	2	2^a^	0
Control	0	26	0	-	-
Total	0	94	2	2	0

^a^Only two specimens with equivocal IHC finding were assessed by real time PCR.

## Discussion

Established data concerning an effective role for certain viral infections in the pathogenesis of breast carcinoma would improve prevention and early detection of this cancer in exposed populations.

We studied the expression of the HCMV IE antigen by immunohistochemistry in tumor cells of invasive breast carcinoma, adjacent non-neoplastic breast tissue, and normal breast parenchyma of reduction mammoplasty specimens in a limited population of Iranian women. The HCMV IE antigen expression was not detected in any of the tumor control and normal control samples. Two tumor specimens showed a double-checked equivocal IHC result. However, these two specimens were found to be negative for HCMV DNA in real-time PCR. Our findings showed no association between CMV infection and invasive breast carcinoma.

The association of HCMV and breast cancer has been investigated in several studies all over the world. Richardson et al*.* [14] from New Zealand used the enzyme immunoassay method in one of their studies, and found evidence of an association between serum CMV IgG level and breast cancer in young women. Taher et al*.* [7] from Sweden demonstrated HCMV IE protein expression in 100% of 73 breast cancer samples using the IHC method and real-time PCR. Harkins et al*.* [19] in the United States also evaluated the surgical biopsy specimens of 38 normal breast samples, 39 breast carcinoma samples, and paired normal breast tissue from 21 breast cancer patients, and demonstrated a higher expression of HCMV antigens in breast cancer compared to normal breast epithelium (97% versus 63%).

Another study by Cox et al*.* [20] in New Zealand showed that the elevation of the serum HCMV IgG prioritizes breast cancer development in a nested case-control study. El-Shinawi et al*.* [21] also reported a significant association between HCMV and breast cancer, with higher serum levels of HCMV IgG in 82% of 28 patients with inflammatory breast carcinoma compared to 65% of 49 patients with non-inflammatory breast carcinoma. Karimi et al. [22] from Iran also detected HCMV DNA in 26 of the 50 samples (58%) of invasive breast carcinoma by using the nested-PCR method in Sanandaj city, Kurdistan Province. Perhaps the different prevalence of HCMV in various geographical areas in Iran may justify the different findings of the Karimi et al. study and ours. Moreover, the different fixation times of tissue samples in various centers, the specificity and sensitivity of the antibody used for the IHC, the variable prevalence of CMV infection in different countries, and time of exposure to the virus in life (early or late) may justify some differences in the research findings.

On the other hand, several studies have failed to show any relationship between HCMV and breast cancer. Utrera-Barillas et al*.* [23] evaluated 27 breast cancer specimens and 20 fibroadenoma samples by quantitative PCR and reported no significant association between HCMV and breast cancer development. Richardson et al*.* [24] also evaluated the CMV IgG levels in plasma (by the enzyme immunoassay method) and checked HCMV DNA in 70 tumor samples using the quantitative PCR method, and found no relationship between HCMV and breast cancer, which went against their previous studies. They suggested some possibilities for these contrasts, such as the limitation of molecular methods and an absence of virus after carcinogenesis, a so-called “hit and run” oncogenesis [24]. Antonsson et al. [11] also failed to detect CMV in breast cancer specimens with the quantitative PCR method in 54 Australian breast cancer tumor specimens.

Sample size, primer designing in PCR based studies, and site of tissue sampling may justify another part of the controversial findings of these studies [25].

## Conclusion

Our results failed to show any relationship between HCMV and breast cancer development. It appears that HCMV does not play an important role in breast cancer carcinogenesis in the Iranian population.

Due to the controversial findings concerning the relationship between HCMV and breast cancer development in several studies in different countries, further studies in this field are mandatory.

## Abbreviations

EBV: Epstein–Barr virus
HCMV: Human cytomegalovirus
HPV: Human Papillomavirus
IE: immediate early antigen
PCR: Polymerase chain reaction
